# Ergothioneine supplementation in people with metabolic syndrome (ErgMS): protocol for a randomised, double-blind, placebo-controlled pilot study

**DOI:** 10.1186/s40814-021-00929-6

**Published:** 2021-10-29

**Authors:** Xiaoying Tian, Giorgia Cioccoloni, Joanna H. Sier, Khalid M. Naseem, James L. Thorne, J. Bernadette Moore

**Affiliations:** 1 School of Food Science & Nutrition, University of Leeds, Leeds, LS2 9JT UK; 2 Leeds Institute of Cardiovascular & Metabolic Medicine, University of Leeds, Leeds, LS2 9JT UK

**Keywords:** Ergothioneine, Metabolic syndrome, Supplementation, Metabolome, Oxidative stress, Inflammation, ALT

## Abstract

**Background:**

Ergothioneine is a naturally occurring metabolite of histidine found in many foods and in high amounts in mushrooms. In vivo, ergothioneine acts as an antioxidant and is widely distributed in most mammalian tissues. While ergothioneine is sold as a dietary supplement for its antioxidant and anti-inflammatory properties, to date there are no published intervention trials examining its health benefits in humans. The aim of this work was to develop a study protocol for a pilot interventional trial that will establish the primary and secondary outcomes, and the power required, for a definitive randomised controlled trial to test the hypothesis that ergothioneine supplementation is beneficial for people with metabolic syndrome.

**Methods:**

We have designed the ErgMS study as a single-centre, randomised, double-blind, placebo-controlled, 3-arm parallel, pilot intervention trial, which aims to supplement participants with either placebo, 5 or 30 mg/day ergothioneine for 12 weeks. Measurements of metabolic syndrome risk factors, serum markers of oxidative stress (lipid peroxidation), inflammation, blood platelet function and liver function will take place at baseline, and after 6 weeks and 12 weeks of supplementation. In addition, we will examine if there are any changes in the serum metabolome in response to ergothioneine supplementation. Linear regression and two-way ANOVA will be utilised to analyse the association between ergothioneine and measured variables.

**Discussion:**

The ErgMS study will be the first study to address the question does ergothioneine supplementation have health benefits for people with metabolic syndrome. Study results will provide preliminary data as to which dose may improve inflammatory markers in adults with metabolic syndrome and will inform dose and primary outcome selection for a definitive randomised controlled trial.

**Trial registration:**

ISRCTN, ISRCTN25890011 Registered February 10^th^, 2021

**Supplementary Information:**

The online version contains supplementary material available at 10.1186/s40814-021-00929-6.

## Introduction

### Background and rationale

Ergothioneine is a naturally occurring metabolite of histidine found in many different foods [1, 2]. Synthesized by bacteria and fungi, ergothioneine is found in particularly high amounts in mushrooms. An amino acid betaine (2-mercapto-histidine trimethylbetaine), ergothioneine has robust cytoprotective properties with roles as a metal chelator as well as an antioxidant, scavenging hydroxyls and other free radicals [3]. As recently reviewed in detail, a growing body of evidence implicates ergothioneine as an important nutrient for healthy ageing and the prevention of a variety of inflammatory diseases, including cardiometabolic diseases [4, 5]. Notably, a long-term prospective cohort study (*n* = 3236 participants with median follow-up of 21.4 years) found that higher plasma levels of ergothioneine were associated with significantly lower risk of coronary disease, cardiovascular mortality and overall mortality (hazard ratios per 1 SD increment of ergothioneine 0.85, 0.79 and 0.86 respectively), as well as a more ‘health-conscious food pattern’ [6].

In vivo, ergothioneine is transported across cell membranes by the solute carrier family 22 member’s 4 and 15 (SLC22A4, SLC22A15) [7–10]. Although many SLC22 family members are non-selective transporters, evidence suggests that SLC22A4 is highly specific for ergothioneine [9, 10]. Widely expressed in most tissues, SLC22A4 is particularly highly expressed in the small intestine where it functions to take ergothioneine up from the diet, and in the kidney where it is presumed to function in renal reabsorption [5]. A pharmacokinetic study of healthy humans taking encapsulated ergothioneine (5 and 25 mg/day) found that ergothioneine is rapidly absorbed and largely retained by the body, with large increases in plasma ergothioneine levels and only minimal increases (< 4%) in urinary excretion observed [11]. Avid absorption and retention of ergothioneine has also been observed in a study in mice, which showed ergothioneine accumulated in liver as well as whole blood [12]. Genetic knockout of SLC22A4 in both mice and zebrafish results in almost complete deficiency of ergothioneine in tissues and increased susceptibility to oxidative stress and inflammation, although organisms remain viable [8, 13].

The existence of a physiologic transporter for ergothioneine along with its antioxidant and cytoprotective roles has raised the question of whether or not ergothioneine should be designated a vitamin [14]. Notably, Ames (2018) has proposed that ergothioneine belongs to a class of ‘longevity vitamins,’ namely dietary compounds not necessarily required for early survival, but that support health and that without which organisms experience accelerated ageing [15]. Supporting this idea are data showing that knockout of SLC22A4 in *Caenorhabditis elegans* increased oxidative damage and reduced lifespan [16], as well as the aforementioned association of high plasma ergothioneine levels with reduced mortality in humans [6]. In a similar vein, Borodina and colleagues (2020) suggest that ergothioneine can be considered a nutraceutical, i.e. a nutrient that when taken at higher amounts than typically found in the diet provides health benefits [4]. Indeed, ergothioneine is already sold as a dietary supplement. The European Food Safety Authority (EFSA) published their safety assessment and scientific opinion for ergothioneine in 2016, determining the no observed adverse effect level (NOAEL) of ergothioneine as 800 mg/kg body weight per day and concluding a supplemental dose of 30 mg/day well within safety margins [17]. Subsequently, the US Food and Drug Administration in 2018 approved ergothioneine as generally recognised (GRAS notice 734) [18].

These regulatory approvals facilitate the needed intervention trials to investigate the potential health benefits of ergothioneine supplementation in humans. In spite of the evidence from pre-clinical models and studies in humans associating low/high blood levels of ergothioneine with disease/health, evidence from randomised controlled trials (RCTs) is lacking. To date, only one study, focused on uptake and pharmacokinetics, has been published examining ergothioneine supplementation in humans [11]. In this placebo-controlled intervention study, 45 participants received either placebo, 5 or 25 mg ergothioneine/day for 7 days and were followed up for an additional 4 weeks; there were no reported adverse effects. While no significant changes in oxidative or inflammatory markers were observed, participants were healthy, the supplementation period was short and the study was likely underpowered to examine these. Currently, a larger and longer-term study is ongoing in Singapore examining the efficacy of ergothioneine (25 mg given 3 times a week for 52 weeks) to delay or reverse cognitive impairment in elderly individuals with mild cognitive impairment [19]. However, a planned study in the USA that aimed to examine the effects of ergothioneine on cognition, mood and sleep in healthy adults has been terminated in the context of COVID [20].

Metabolic syndrome is a cluster of risk factors associated with type 2 diabetes and cardiovascular and atherothrombotic diseases [21]. Oxidative stress plays an important role in the pathogenesis of metabolic syndrome and its downstream morbidities [22]. Given the lack of data in humans, in order to address the hypothesis that ergothioneine supplementation may reduce markers of systemic and cellular oxidative stress and inflammation in people with metabolic syndrome, a pilot study is necessary to establish the primary and secondary outcomes and power required for a definitive RCT. The ErgMS study aims to establish these and will be the first study to investigate potential health benefits of ergothioneine in people with metabolic syndrome.

### Study aims

The overall aims of this trial are to determine the feasibility of supplementing people with metabolic syndrome with ergothioneine for 12 weeks, and to establish primary and secondary outcomes and the power required for a definitive RCT to establish the benefit of ergothioneine on markers of oxidative stress, liver damage, inflammation and metabolic syndrome risk factors among people with metabolic syndrome.

### Study objectives

1. To assess feasibility of recruiting and supplementing people with metabolic syndrome with ergothioneine for 12 weeks.
2. To investigate if any changes in serum markers of oxidative stress (lipid peroxidation), inflammation and liver function can be observed in response to ergothioneine supplementation.
3. To examine if any changes in oxidative and inflammatory stress in blood immune cells can be observed in response to ergothioneine supplementation.
4. To assess if there are any changes in metabolic syndrome risk factors or in the serum metabolome in response to ergothioneine supplementation.
5. To use the data to determine dose of ergothioneine and participant numbers needed to inform a primary outcome for a definitive randomised controlled trial.

### Study design

The ErgMS study has been designed as a single-centre randomised, double-blind, placebo-controlled, 3-arm parallel, pilot interventional trial.

**Fig. 1:**
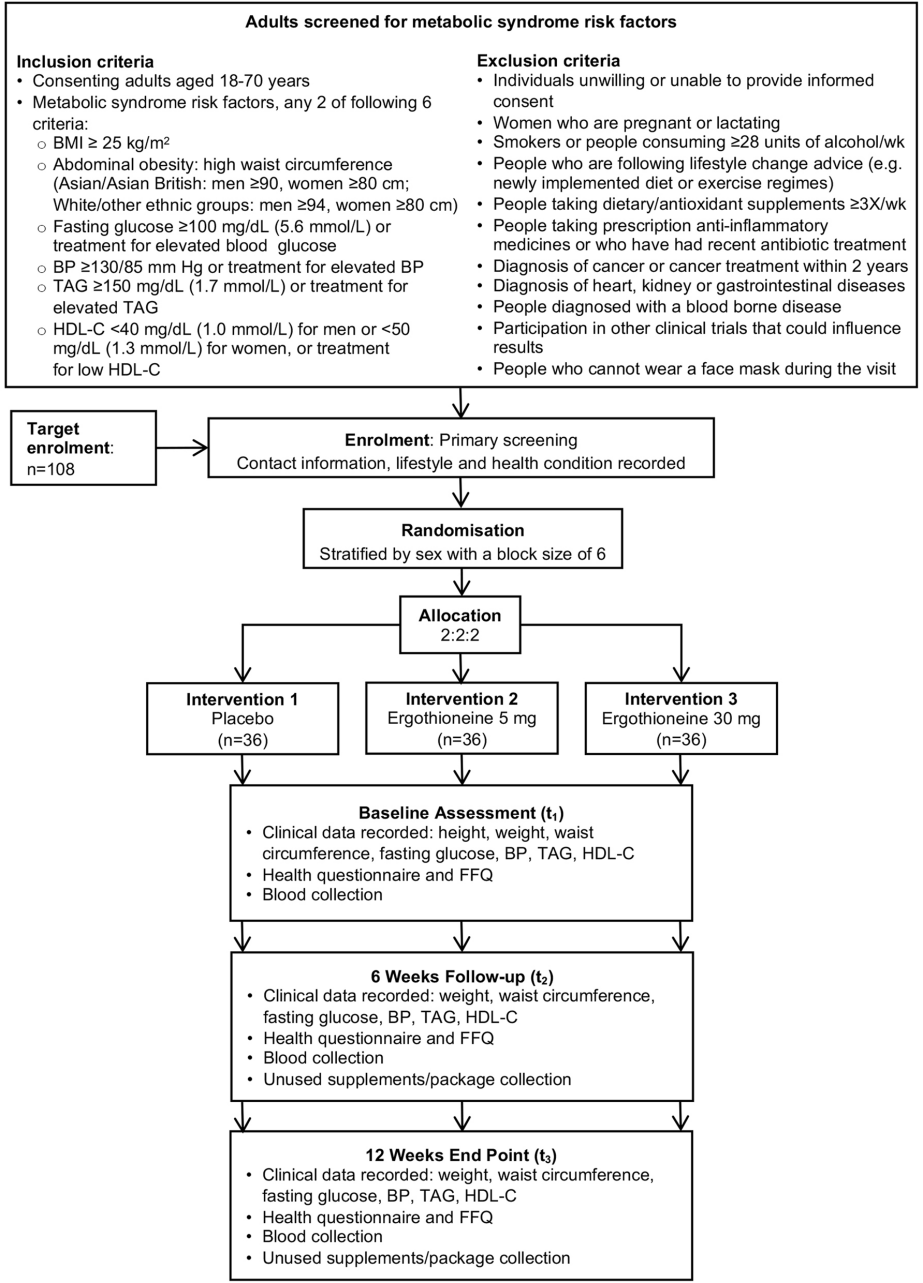
ErgMS Study design. *BMI* body mass index, *BP* blood pressure, *FFQ* food frequency questionnaire, *TAG* triacylglyceride, *HDL*-*C* high-density lipoprotein-cholesterol

Consenting participants with metabolic syndrome risk factors will be randomised in a double-blind fashion to one of three parallel arms to receive either placebo (0 mg ergothioneine), 5 mg ergothioneine or 30 mg ergothioneine for consumption as daily capsules (one per day) for 12 weeks (Fig. 1). The dose of 30 mg is well within the NOAEL of 800 mg/kg body weight set by EFSA [17]. Participants will give blood samples and undergo anthropometric measurements at three timepoints, baseline, 6 weeks and 12 weeks (Fig. 1). The ErgMS protocol was designed following the Standard Protocol Items: Recommendations for Interventional Trials (SPIRIT) guidance [23] and has been prospectively registered with the ISCRCTN registry, reference: ISRCTN25890011 10.1186/ISRCTN25890011.

## Methods

### Study setting

The study will be conducted at the School of Food Science and Nutrition at the University of Leeds, in Leeds, West Yorkshire, the UK.

### Eligibility criteria

#### Inclusion criteria

Consenting participants must be 18–70 years old and have risk of metabolic syndrome, defined by presenting with at least two of the six following criteria; with cut-offs based on the 2009 consensus definition of metabolic syndrome and population-specific thresholds for defining excess central adiposity [21]:

1. BMI ≥ 25 kg/m^2^;
2. Abdominal obesity: high waist circumference (Asian/Asian British—men ≥ 90 cm; women ≥ 80 cm; White and all other ethnic groups—men ≥ 94 cm, women ≥ 80 cm);
3. Fasting glucose ≥ 100 mg/dl (5.6 mmol/l) or treatment for elevated blood glucose;
4. Blood pressure ≥ 130/85 mmHg or treatment for elevated blood pressure;
5. Triacylglycerides (TAG) ≥ 150 mg/dl (1.7 mmol/l) or treatment for elevated TAG;
6. Cholesterol-high density lipoprotein (HDL-C) < 40 mg/dl (1.0 mmol/l) for male or < 50 mg/dl (1.3 mmol/l) for female or treatment for low HDL-C.

#### Exclusion criteria

1. People who are unwilling or unable to provide informed consent;
2. Women who are pregnant or lactating;
3. Smokers or people who consume ≥ 28 units of alcohol per week (28 units = ~ 10 medium glasses of wine (175 ml) or ~ 10 pints of beer/cider);
4. People following lifestyle change advice, e.g. newly implemented diet or exercise regime (≥ 150 min/week moderate aerobic exercise or ≥ 75 min/week vigorous aerobic exercise) aimed at weight loss;
5. People who have regularly (≥ 3 times/week) taken dietary/antioxidant supplements within the last 4 months;
6. People taking prescription anti-inflammatory medicines (occasional aspirin, paracetamol, ibuprofen use acceptable);
7. Antibiotic treatment within last month or 3 courses within the last 6 months;
8. Diagnosis of cancer or end of cancer treatment within 2 years;
9. Diagnosis of heart, kidney or gastrointestinal diseases or people who have had surgical treatment for obesity;
10. People diagnosed with a blood borne disease (HepB, HIV etc.);
11. Participation in other clinical trials that may influence outcomes;
12. In the context of COVID-19 safe procedures, people who cannot wear a face mask during the visit

### Consent

Electronic copies of the participant information leaflet and consent form will be sent to any potential participants when they show interest in the ErgMS study and provide their email address requesting more information. Preliminary screening will be done online and an online consent form must be signed before the screening questionnaire will be released. At the baseline visit, informed consent will be confirmed with electronic written consent taken prospectively from the participants by the site study team in advance of collecting any data. The consent form will ask volunteers to confirm their understanding that the samples and data collected will be used to support other research in the future, and may be shared anonymously with other researchers. The form will also give potential participants the option to agree to be contacted for future studies. Only adults (> 18 years old) who have the capacity to provide consent to participate will be included. The ErgMS study will be conducted according to the principles of Good Clinical Practice, and to the ethical principles that have their origin in the Declaration of Helsinki.

### Interventions

Consenting participants will be randomised in a double-blind fashion to one of three intervention groups that will receive either placebo (0 mg ergothioneine), 5 mg ergothioneine or 30 mg ergothioneine for consumption as daily capsules (one per day) for 12 weeks (Fig. 1). Both placebo and ergothioneine supplement capsules will be manufactured by Tetrahedron (Paris, France). The placebo capsules will have the same shape, colour, weight and packaging as the ergothioneine capsules. Participants can withdraw from the study at any time if they want to withdraw without reasons by contacting the researcher. Data already collected from any withdrawals will be kept in the dataset and used in intention-to-treat analysis.

To promote adherence, participants will be sent periodic reminder/enquiry texts before each visit and will be informed of their clinical parameters at each visit and the final results after the trial data has been analysed and unblinded. Supplementation compliance will be measured both by capsule counting (participants will be asked to return package and untaken supplements at 6 weeks and 12 weeks) and the measurement of ergothioneine in plasma by high-performance liquid chromatography (HPLC) at baseline, 6 weeks and 12 weeks.

Related to the exclusion criteria, participants will be asked not to start taking dietary/antioxidant supplements, implement a new diet/fitness regime or take prescription anti-inflammatory medicines (occasional aspirin, paracetamol, ibuprofen use acceptable) for the duration of the trial.

### Outcomes

#### Primary feasibility outcomes

1. Recruitment and completion will be measured in the numbers of participants enrolling and completing all study visits;
2. Supplementation compliance will be measured both by capsule counting (participants returning packaging and untaken supplements) and the measurement of ergothioneine in plasma by HPLC at baseline, 6 weeks and 12 weeks.

#### Secondary exploratory outcomes

At baseline, 6 weeks and 12 weeks, we will measure:

1. A primary serum marker of oxidative stress (specifically, lipid peroxidation), malondialdehyde (MDA), which we hypothesize could be the future primary outcome for powering a future definitive trial;
2. Serum markers of inflammation (tumour necrosis factor-alpha (TNF-α) protein, nuclear factor erythroid 2-related factor 2 (Nrf2) protein, c-reactive protein (CRP), soluble CD36, NADPH oxidase 4 (NOX4) mRNA) and liver function (ALT);
3. The activation status of blood platelets, platelet reactive oxygen species (ROS) generation and inflammatory platelet-leukocyte aggregates;
4. Metabolic syndrome risk factors: body weight (for BMI calculation, height will be measured at baseline only) and waist circumference, blood pressure, triacylglyceride (TAG), high-density lipoprotein (HDL) cholesterol and fasting glucose;
5. Serum metabolites.

### Participant timeline

The participant timeline and schedule of the intervention is outlined in Table 1 in accordance with SPIRIT guidelines.

**Table 1:**
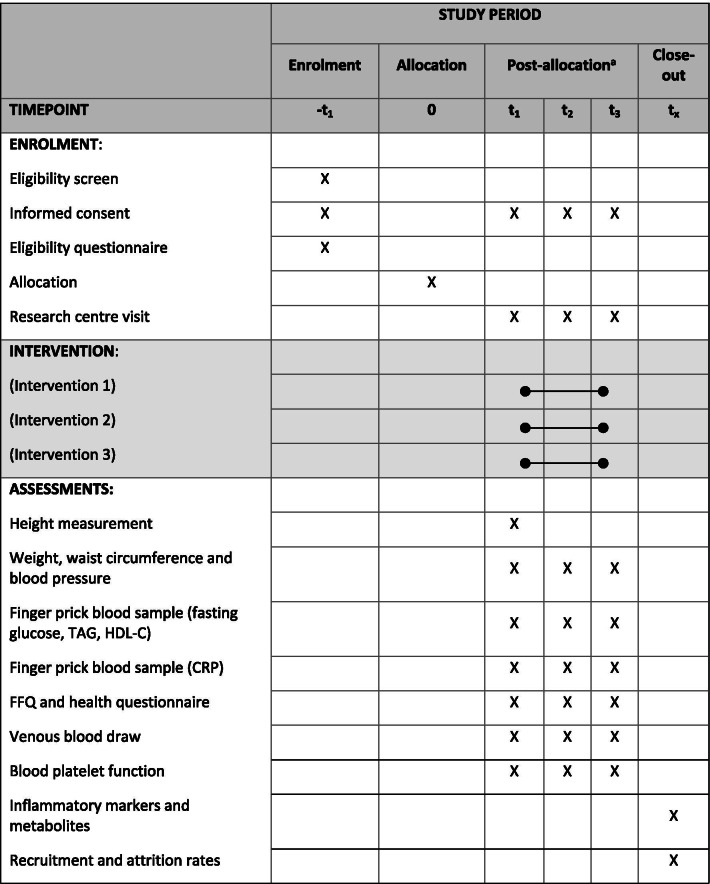
Participant timeline from initial screening to end of study

^a^*t*_1_, baseline; *t*_2_, 6 weeks, *t*_3_ 12 weeks

### Sample size

As a consequence of a complete lack of data on which to base either a primary outcome or a formal power calculation, and in line with the appropriate CONSORT extension [24], a primary aim of this pilot trial is to generate the data to inform the design of a definitive trial. The decision to recruit a total 108 participants was a pragmatic one based both on statistical arguments for sample size in pilot/feasibly trials and the need to balance maximizing precision while minimizing the impacts of size on resources, time and costs [25, 26]. With a goal of 90 measured participants (30 per group), we aim to recruit 108 participants to allow for 20% drop out per group. This number is in line with previous calculations suggesting between 25 and 35 participants per group will typically provide sufficient power to determine the effect size for a sample size calculation without being wasteful of resources [25–27].

### Recruitment

We will recruit participants in several ways. People with metabolic syndrome who participated in a previous study in our School (the PATHWAY-27 study [28]), and consented to be contacted again for other research studies, will be contacted by phone or email to ask if they are interested in taking part in another intervention trial. We will also advertise the study broadly through conventional and social media and via multiple relevant charities (e.g. British Heart Foundation and Diabetes UK). We aim to achieve Health Research Authority approval, which will also permit recruitment through general practitioners’ offices. Based on experience with the PATHWAY-27 study [28], we anticipate recruitment and running of the trial to take 2 years. In the context of unforeseen risk events such as COVID19 or low recruitment rates, given our block design this time can be extended.

### Assignment of interventions

#### Allocation

While enrolment will be done by the study team, the randomisation schedule and allocation will be carried out by a collaborator who will not be involved in any other part of this trial and who will also be blinded to the dose allocation (Interventions ‘1’, ‘2’ or ‘3’; Fig. 1) designated by the manufacturer and provided in a sealed envelope. Randomisation and group allocation will be done based on predefined random allocation lists using simple stratification for sex (one each for males and females), with a block size of 6 and allocation list of 2:2:2; aiming to recruit 108 participants for *n* = 36 in each group.

#### Blinding

Both participants and researchers, including research team members and the collaborator, will be blinded to the dose allocation. The collaborator will maintain the dose allocation lists matching screening IDs to randomisation IDs, along with the manufacturer’s sealed envelope for the duration of the trial. This envelope will only be opened before study completion in the case of a serious adverse event. To test the success of blinding, 2 external questions will be added in the final visit of the health questionnaire—‘What dose of ergothioneine did you think you were taking during this period?’ & ‘Why?’.

### Data collection, management and analysis

#### Data collection methods

Participants will give fasted blood samples and undergo anthropometric measurements at three timepoints, baseline, 6 weeks and 12 weeks. All the clinical data (metabolic syndrome risk factors) will be recorded in clinical research files. As detailed in the data management plan below, these will be scanned and copies saved electronically as well as in physical format. At each visit, participants will be asked to complete a brief online health questionnaire and an online food frequency questionnaire (FFQ) adapted from our validated short (45-item) FFQ [29] to include food items rich in ergothioneine.

#### Blood sample collection

Minimal amounts of blood will be taken from participants to limit the burden of bleeding. At three timepoints: baseline (before starting capsule supplementation), 6 weeks follow-up and end of the study, we will ask to take a finger prick blood sample to measure participants blood TAG, HDL cholesterol, fasting glucose and CRP. In addition, participants will be asked to provide 15 ml of blood sample for measurement of platelet function, inflammatory biomarkers and metabolites. Fifteen milliliters of blood donation is safe for adults and venepuncture will be done by a trained phlebotomist to ensure that distress during blood collection will be minimal and that correct procedures for collection, processing and storage are followed. Five milliliters of whole blood will be immediately used for platelet function assays using multicolour flow cytometry assessment of surface markers of platelet activation, platelets subsets, platelet ROS generation and platelet-leukocyte aggregates [30]. Platelet function will be addressed under basal conditions in order to evaluate activation status of circulating platelets and after stimulation with known agonists to examine if their propensity for activation is altered. The remaining 10 ml will be centrifuged for the collection of acellular serum, aliquoted and stored at − 80 °C for future laboratory analysis. Stored serum will be compliant (i.e. exempt from regulation) with the 2004 Human Tissue Act.

#### Metabolic syndrome risk factors

Height will be measured with a stadiometer at baseline. Bodyweight, blood pressure, waist circumference will be measured with a beam scale, blood pressure monitor and tape respectively at baseline, 6 weeks and 12 weeks. TAG, HDL-C and fasting glucose will be measured in whole blood using a rapid point-of-care multi-assay blood analyser (Afinion 2 analyser; Abbott, UK) at baseline, 6 weeks and 12 weeks.

#### Biomarkers assessment

CRP will also be measured from a finger prick of whole blood by a rapid point-of-care multi-assay blood analyser. MDA will be measured in purified serum samples by HPLC. The TNF-α and Nrf2 proteins will be measured in purified serum samples using enzyme-linked immunosorbent assay (ELISA) kits. NOX4 mRNA expression will be measured in purified serum samples by quantitative polymerase chain reaction. ALT activity will be measured in serum samples using a commercial assay kit.

#### Metabolite assessment

 Ergothioneine and its metabolites (hercynine, ergothioneine sulfonate and *S*-methyl ergothioneine), as well as other metabolites will be measured in serum by liquid chromatography-mass spectrometry (LC-MS).

#### Online questionnaires

Online health questionnaire and FFQs will be used to record the health condition, dietary habit and quality at baseline (visit 1), 6 weeks (visit 2) and end of the study (12 weeks, visit 3).

### Data management, confidentiality and access

All data will be managed through a Microsoft Access database with data entered using encrypted, two-factor authentication protected, computers owned by the University of Leeds. Electronic data will be stored on the University of Leeds SAN (Storage Area Network), which comprises enterprise level disk storage and file servers located in physically secure data centres with appropriate fire suppression equipment and daily/monthly/long-term back up protocols. The SAN is located behind the University’s Institutional firewall to protect against external attacks.

Physical format clinical research files (used to collect clinical data during visits; template available in appendix of our approved ethics application) will be scanned and copies saved electronically as well as in physical format in locked cabinet in the chief investigator’s secure office. Data entry from clinical research files will be confirmed independently by second investigator. Questionnaires (primary eligibility screening questionnaire, health questionnaires, FFQs) will be collected in a digital format.

Data will be collected anonymised with only participants’ randomisation number and date of birth recorded on the data collection tools. Anonymised data will be shared, published and used in line with the University of Leeds Research Data Management Policy. Any personal details, identifiable or sensitive information will not appear in any publications. The data will be retained for at least 2 years after publication. Only research team members will have access to the data.

### Statistical methods

Descriptive statistics will be used initially to present the basic features of the data, with data presented as mean/SD or percentage used as appropriate in response to normality testing using (Table 2). Linear regression and two-way ANOVA will be performed to analyse the associations between variables (Table 3). The significance level will be set at 5% and 95% confidence intervals will be presented. Intention-to-treat analysis will be performed to compare the difference with per-protocol analysis. Basic analyses will be performed in GraphPad Prism. Multivariate linear regression will be done either in STATA or the R environment in consultation with statistician/mathematical modeller as required.

**Table 2:** Descriptive characteristics

	Mean ± SD or %
Age (years)	
Ethnicity	
Asian/Asian British	
White and all other ethnic groups	
Sex	
Male	
Female	
Smokers	
Alcohol intake (units/week)

**Table 3:** ErgMS measurements and schedule

Measurement	Study visit		
	Baseline	6 wks	12 wks
Primary			
Recruitment			
Completion			
Supplementation compliance			
Secondary			
Platelet function			
MDA			
ALT			
TNF-α			
Nrf2			
CRP			
Fasting glucose			
TAG			
HDL-C			
Weight			
BMI			
WC			
Systolic BP			
Diastolic BP			

*ALT* Alanine transaminase, *BMI* Body mass index, *BP* Blood pressure, *CRP* C-reactive protein, *HDL*-*C* High-density lipoprotein-cholesterol, *MDA* Malondialdehyde, *Nrf2* Nuclear factor erythroid 2-related factor 2, *TAG* Triacylglyceride, *TNF*-*α* Tumour necrosis factor-alpha, *WC* Waist circumference, *wks* Weeks

### Monitoring

The ErgMS trial management group will be comprised of the Chief (JBM) and lead investigators (XT, JS, JT) and will be responsible for the day-to-day running and management of the trial. As ergothioneine is an amino acid normally found in food and the doses of ergothioneine being tested (5 or 30 mg/day) are well below the safety limit (800 mg/kg bodyweight per day—e.g. a total of 56,000 mg/day for a 70 kg adult), the risk of harm or adverse event for adult participants with metabolic syndrome is considered low. Ergothioneine has minimal allergy risk determined by European Food Safety Authority [17]. Participants will be asked to inform the researcher if they feel unwell at any point during the study and their general health will be assessed through the health questionnaire at the 6- and 12-week visits.

Any participant reported adverse events will be recorded by the member of the trial management group in our adverse event reporting form and immediately report it to the Chief Investigator and assessed for causality. As necessary, the participant will be advised to consult their GP or relevant healthcare professional. In light of the relatively low risk study population with a noncritical indication (adults with metabolic syndrome), use of a food supplement with minimal risk, and short study duration, the ErgMS study does not have an independent data monitoring committee. In the unlikely event of a serious adverse event (SAE), the participant’s allocated intervention will be unblinded and the SAE and allocation will be reported to the Research Ethics Committee and the supplement manufacturer. Although there are minimal risks anticipated for this study, any adverse event or serious adverse event will be described according to its relatedness with the interventions, for both the total sample and by intervention arms, in the final study report.

### Protocol amendments

Any modifications of the protocol that may affect the conduct of the study, participant recruitment, the information provided to participants or the data analysis will be submitted to the University of Leeds Research Ethics Committee as an amendment and communicated to ISRCTN after approval. As necessary, any changes affecting participants currently enrolled will be communicated to participants after approval and being applied to the study.

### Dissemination plans

Regardless of the magnitude or direction of effect, the results will be disseminated in a timely fashion through presentation first at scientific conferences and then via publication in open access, peer-reviewed journals. In addition, progress reports and results will be available on the trial website and communicated via social media to a wider audience. The full protocol, and ultimately the anonymised dataset and any relevant statistical code, will be deposited in the Leeds Research Data Repository and available open access.

## Discussion

Ergothioneine is a dietary nutrient that is sold as a dietary supplement for its antioxidant and anti-inflammatory properties. However, to date, there are no published intervention trials examining its efficacy in humans. Therefore, along with examining feasibility, the ErgMS study aims to establish primary and secondary outcomes, and the power required, for a definitive randomised controlled trial to test the hypothesis that ergothioneine supplementation is beneficial for people with metabolic syndrome.

A significant body of preclinical data has demonstrated antioxidant and anti-inflammatory activities of ergothioneine that are relevant to the prevention of cardiometabolic diseases [4, 5]. These data underpinned our decision to first investigate ergothioneine in people with metabolic syndrome, who are at risk of developing diabetes and non-alcoholic fatty liver disease (NAFLD), as well as cardiovascular and atherothrombotic diseases [21]. Considered the hepatic manifestation of the metabolic syndrome [31], NAFLD involves chronic oxidative metabolism, lipid peroxidation and inflammation that can progress to liver fibrosis [32]. Interestingly, ergothioneine has been shown to be protective in both animal models of NAFLD [33] and liver fibrosis [34], informing our measurement of MDA, a primary serum marker of lipid peroxidation, as well as ALT, a marker of liver function in this study. Similarly, several of the inflammatory markers we intend to measure have been shown to be modulated in protective fashion by ergothioneine in preclinical models; e.g. TNF-α [35], Nrf2 [36] and NOX4 [34]. In the context of COVID-19, a possible factor affecting measures of inflammation might be vaccination timing, and this information will be collected from participants and examined as a potential confounder.

Alterations in platelet function, including platelet hyperaggregability and hyperactivation is commonly seen in people with metabolic syndrome and contributes fundamentally to the increased risk of atherothrombotic diseases [37, 38]. Oxidative stress plays a critical role in platelet hyperactivity, with platelet activation driven by ROS present both in the vasculature and generated by platelets themselves. Dietary antioxidant intakes are inversely associated with cardiovascular and atherothrombotic diseases [39], and multiple dietary supplements and nutraceuticals have been shown to impact platelet function [40–42]. Although the antioxidant activities of ergothioneine in scavenging hydroxyls and other free radicals is well documented [3], to date the role of ergothioneine in human platelet function has not been explored. Therefore in the ErgMS study, we will assess platelet function using multicolour flow cytometry to measure surface markers of platelet activation, platelets subsets, platelet ROS generation and platelet-leukocyte aggregates [30].

Potential limitations to this study exist, including the fact that there were no data on which to base the length of supplementation period for ergothioneine. Therefore, the decision to supplement for 12 weeks, with measurements also taken at 6 weeks was a pragmatic one. Although serum MDA has been shown to respond to intervention with other dietary supplements within 12 weeks [43, 44], these are not directly comparable to ergothioneine. Likewise, there were no data on which to base the dose of ergothioneine for supplementing people with metabolic syndrome, and pragmatically the decision was based to supplement with the amounts (5 mg and 30 mg) commonly available and well within the safety margins defined by EFSA [17]. In the only human study published to date, where healthy participants received either placebo, 5 or 25 mg ergothioneine/day for 7 days, a dose-dependent effect on levels of ergothioneine in plasma, urine and whole blood were observed [11]. However, data from mice orally administered either saline, 35 or 70 mg/kg ergothioneine per day for 1, 7 or 28 days, suggests saturation kinetics exist for ergothioneine uptake and accumulation in animal tissues [12]. Therefore, it is difficult to predict whether the lower or higher dose of ergothioneine may be more beneficial over 12 weeks for people with metabolic syndrome.

An additional potential implication from the ErgMS design is that while the exclusion criteria reflect our objective of focusing on metabolic syndrome and excluding other inflammatory conditions, the large number of exclusion criteria may result in a longer recruitment period. Nonetheless, the ErgMS study will be the first study to address the question does ergothioneine supplementation have health benefits for people with metabolic syndrome. Study results will provide novel, preliminary data as to which dose may improve inflammatory markers in adults with metabolic syndrome, and inform dose and primary outcome selection for a definitive RCT.

## Supplementary Information

**Additional file 1.**

## Data Availability

Not applicable.

## Abbreviations

ALT: Alanine transaminase
CRP: C-reactive protein
EFSA: European Food Safety Authority
ELISA: Enzyme-linked immunosorbent assay
FFQ: Food Frequency Questionnaire
HDL: High-density lipoprotein
HPLC: High-performance liquid chromatography
LC-MS: Liquid chromatography-mass spectrometry
MDA: Malondialdehyde
NOX4: NADPH oxidase 4
Nrf2: Nuclear factor erythroid 2-related factor 2
RCT: Randomised controlled trial
ROS: Reactive oxygen species
SLC22A4: Solute carrier family 22 member 4
TAG: Triacylglyceride
TNF-α: Tumour necrosis factor-alpha
