# Supplementary material for: Ergothioneine supplementation in people with metabolic syndrome (ErgMS): protocol for a randomised, double-blind, placebo-controlled pilot study
Source: Pilot Feasibility Stud. 2021 Oct 29;7:193. doi: 10.1186/s40814-021-00929-6 (PMC8555363; doi:10.1186/s40814-021-00929-6)
Supplement: Supplementary file 1 — Additional file 1. [file 40814_2021_929_MOESM1_ESM.docx]

Investigating the potential health benefits of ergothioneine supplementation in people with metabolic syndrome

**Appendices to University of Leeds Ethics Application**

Xiaoying Tian, James L Thorne, J Bernadette Moore

Table of Contents

1. [Primary Eligibility Phone Screening 1](#_Toc66101779)
2. [Primary Eligibility Email Screening 6](#_Toc66101780)
3. [Poster 8](#_Toc66101781)
4. [Participant Information Sheet 9](#_Toc66101782)
5. [Consent Form 13](#_Toc66101783)
6. [Primary Eligibility Questionnaire 14](#_Toc66101784)
7. [Visit 1 Baseline Clinical Research File 18](#_Toc66101785)
8. [Visit 2 Follow-up 6 Weeks Clinical Research File 20](#_Toc66101786)
9. [Visit 3 Endpoint Clinical Research File 21](#_Toc66101787)
10. [Food Frequency Questionnaire 22](#_Toc66101788)
11. [Food Frequency Questionnaire (Erg focused) 27](#_Toc66101789)
12. [Visit 1 Health Questionnaire 30](#_Toc66101790)
13. [Visit 2 Health Questionnaire 32](#_Toc66101791)
14. [Visit 3 Health Questionnaire 34](#_Toc66101792)
15. [Adverse Events /Serious Adverse Events Form 36](#_Toc66101793)


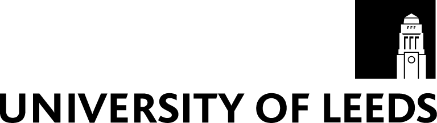


# Primary Eligibility Phone Screening

Thank you for your interest in taking part in this study to investigate the potential benefits of ergothioneine in people with metabolic syndrome.

In order to help us to determine whether you are eligible to take part, we would like to take some general information from you and ask some questions about your lifestyle and health.

| **Contact details** | |
| --- | --- |
| First name |  |
| Surname |  |
| Telephone number |  |
| Mobile number |  |
| Email address |  |
| Home address |  |

| **General information** | | |
| --- | --- | --- |
| Gender | Male | Female |
| Date of birth |  | <18 / >70 y = exclude |
| Height | (cm / metres / feet & inches) | |
| Weight | (kg / lbs / stones & lbs) | |
| Waist circumference | (cm / feet & inches) | |
| Note: this is a measurement around the fullest point of your waist. If you do not have a tape, please write your dress/trousers size starting with ‘dress size:’ or ‘trousers size:’. | | |

| **Lifestyle** | | | |
| --- | --- | --- | --- |
| Are you pregnant, lactating or trying to conceive? | YES | NO | YES = exclude |
| Do you smoke? | YES | NO | YES = exclude |
| Do you drink alcohol? | YES | NO |  |
| **If yes,** how much do you drink alcohol per week? |  | | ≥ 28 units per week (28 units = ~10 medium glasses wine (175mlL) or ~10 pints of beer/cider) = exclude |
| As a guide, 1 pint of beer / 1½ bottles of lager / 1 medium (175 mL) glass of wine = 2-3 units | | | |
| Have you taken or are you currently taking any vitamin or herbal supplements? | YES | NO |  |
| **if yes**, how often/how many times a week or month?  If not currently, when was the last time you took a supplement |  | |  |
| **If yes**, please give details. What supplements/ how often. |  | | Yes = check if relevant.  If antioxidative supplement within 4 months = exclude |
| Have you gained or lost weight more than 2-3kg in last 3 months? | YES | NO |  |
| Are you currently on a specific diet or trying to lose weight? | YES | NO | YES = exclude |
| Do you regularly exercise? | YES | NO |  |
| **If yes,** how vigorously, how often and how long? |  | | ≥150 min/week moderate aerobic exercise or ≥75 min/week vigorous aerobic exercise = exclude |
| Are you currently following advice to change your lifestyle (e.g. newly taking up exercise)? | YES | NO | YES = exclude |

| **Health condition** | | | | | |
| --- | --- | --- | --- | --- | --- |
| Have you had a covid vaccine? | YES | | NO | |  |
| **If yes**, which type? |  | | | | |
| **If yes**, how many doses have you had? | 1 | 2 | | Other |  |
| what date was last dose administered? |  | | | |  |
| Have you ever been told that you have any of the following? | | | | | |
| High blood pressure (hypertension) | YES | | NO | |  |
| High blood triglycerides | YES | | NO | |  |
| High blood glucose | YES | | NO | |  |
| Altered blood cholesterol (high LDL or low HDL) | YES | | NO | |  |
| Do you have any diseases in your family history? | YES | | NO | | YES = check if relevant |
| **If yes**, please give details. |  | | | |  |
| Have you been diagnosed with any of the following? | | | | | |
| Liver disease | YES | | NO | | YES = check if relevant |
| **If yes**, please give details. |  | |  | |  |
| Diabetes | YES | | NO | |  |
| Heart disease | YES | | NO | | YES = exclude |
| Intestinal disorders (Crohn’s disease, short bowel syndrome, pancreatic insufficiency, cystic fibrosis, tropical sprue, whipple’s disease, chronic pancreatitis, gastrojejunostomy, surgical treatment for obesity, cholestasis, biliary atresia, parasite infections) | YES | | NO | | YES = exclude |
| Kidney disease | YES | | NO | | YES = exclude |
| Blood borne disease (HepB, HIV etc.) | YES | | NO | | YES = exclude |
| Cancer | YES | | NO | |  |
| **If yes**, when did treatment end? |  | | | | End of treatment <2 y = exclude |
| Any other disease in the last 2 years? | YES | | NO | | YES = check if relevant |
| **If yes**, please give details. |  | | | |  |
| Are you currently taking or regularly taking any prescription or non-prescription medicine? | YES | | NO | | YES = check if relevant |
| **If yes**, please give details**.** |  | | | | anti-inflammation = exclude (occasional aspirin, paracetamol, ibuprofen use acceptable) |
| Are you taking medication for diabetes or high blood pressure? | YES | | NO | |  |
| Have you ever had antibiotic treatment? | YES | | NO | |  |
| **If yes**, what is the first time you receiving the treatment? When did treatment end? |  | | | | Receiving treatment < 1 month, or receiving 3 courses treatment < 6 months = exclude |

| **Ability** | | | |
| --- | --- | --- | --- |
| Are you currently taking part in any other clinical trial? | YES | NO | YES = exclude |
| Are you able to come to the University of Leeds on 3 time points during the 12 weeks (before start, 6 weeks, 12 weeks)? | YES | NO | NO = exclude |
| Are you able to make your own decisions and **not** under the care of the state, by judicial or administrative decision? | YES | NO | NO = exclude |

Thank you for answering these questions.

**Responses**

**Eligible**

I can confirm that you are eligible to take part in the trial and I would like to invite you to come to the University for a before-start examination. This will involve measuring your height, weight, waist circumference and blood pressure, and taking some blood for measurements of blood sugar and cholesterol.

If you would still like to take part, would you like to make an appointment now?

| Date |  | Time |  |
| --- | --- | --- | --- |

**YES**

I will send you a text message to confirm your appointment with a link to our Participant Information Sheet explaining details about the study. If you want to withdraw or change your appointment at any time, please feel free to contact me. Thank you for your time answering these questions.

| Date |  | Time |  |
| --- | --- | --- | --- |

**Not Sure, please call me later.**

Thank you for your time answering these questions.

**NO, I don't want to take part.**

Thank you for your time answering these questions.

**Not eligible**

I'm sorry you meet one or more our exclusion criteria. I am not able to invite you to take part.

Thank you for your time answering these questions.


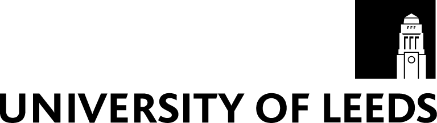


# Primary Eligibility Email Screening

Dear Dr/Mr/Ms xxx,

Thank you for your interest in taking part in this study to investigate the potential benefits of ergothioneine in people with metabolic syndrome. In order to help us to determine whether you are eligible to take part, we need to ask for some general information from you and ask some questions about your lifestyle and health.

Please find attached our Participant Information Sheet and our Questionnaire:

I am happy to go through these on a phone call if necessary. Please feel free to contact me if you have any questions.

Thank you very much for taking your time answering these questions and your help with our study.

Looking forward to your reply.

Best wishes,

Xiaoying

Xiaoying Tian

Postgraduate Researcher

University of Leeds

School of Food Science & Nutrition

2.36 Chemistry Building

LS2 9JT

**Responses**

Dear Dr/Mr/Mrs/Miss xxx,

Thank you for your interest in taking part in this study.

**If eligible:**

I can confirm that you are eligible to take part in the trial and I would like to invite you to come to the University of Leeds for a before-start examination. This will involve measuring your height, weight, waist circumference and blood pressure and taking some blood for blood sugar and cholesterol measurements.

If you would still like to take part, please reply with the date for a morning (8am-12am Monday-Friday for fasting measurements) you would prefer to make an appointment.

Looking forward to your reply.

**If not eligible**

I'm sorry you meet one or more our exclusion criteria. I am not able to invite you to take part the following stage.

Thank you very much for your interest and taking time answering these questions.

Best wishes,

Xiaoying

Xiaoying Tian

Postgraduate Researcher

University of Leeds

School of Food Science & Nutrition

2.36 Chemistry Building

LS2 9JT


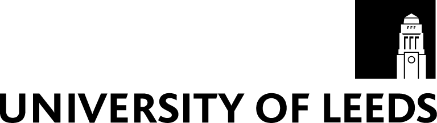


#
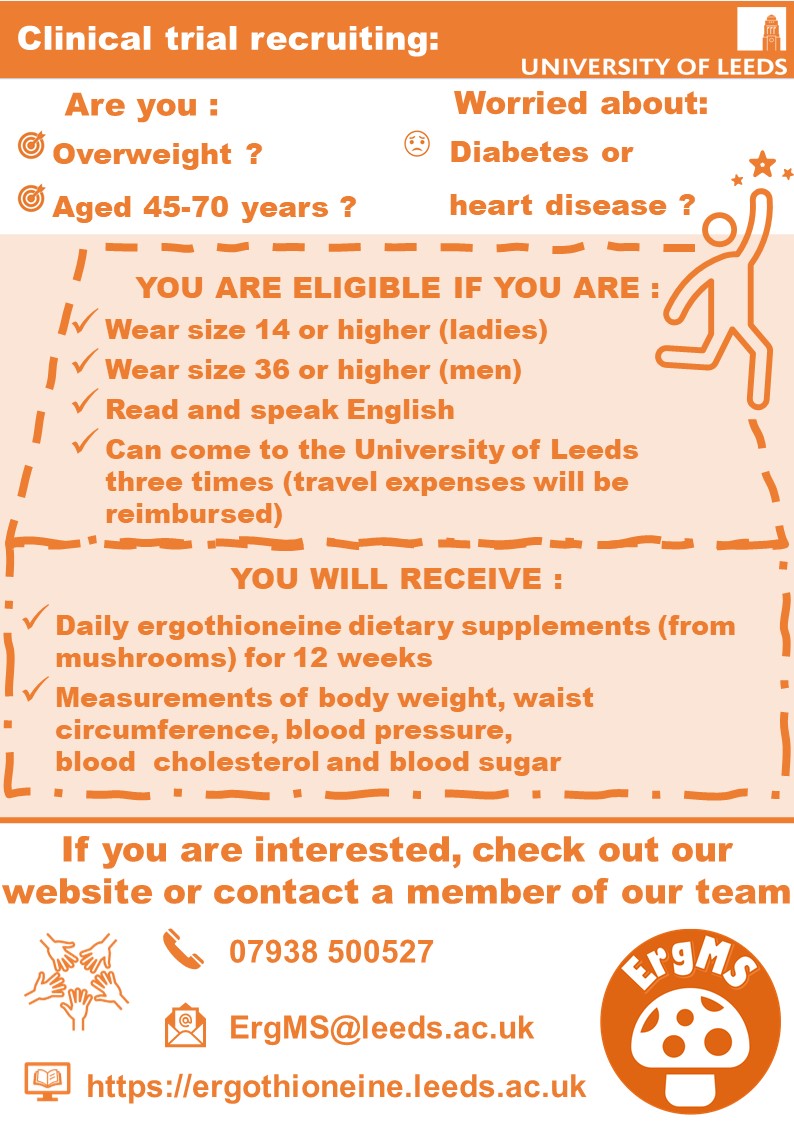
Poster


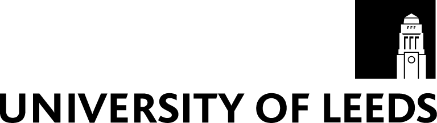


# Participant Information Sheet

**The title of the research project:**

Investigating the potential health benefits of ergothioneine supplementation in people with metabolic syndrome (ErgMS)

You are invited to take part in a research project at the University of Leeds. Before you decide to take part, it is important for you to understand the purpose of the study and what it will involve. Please take time to read the following information carefully. If you have any questions or would like more information, please feel free to ask us.

**What is the purpose of the study?**

The aim of our study is to investigate the effects of 12 weeks ergothioneine supplementation in people with metabolic syndrome. We will assess changes in indicators of metabolic syndrome, oxidative stress, inflammation and liver function.

**Why have I been chosen?**

You have been invited to take part in the study because we are looking for adults with risks of metabolic syndrome. Metabolic syndrome is defined as a cluster of risk factors for cardiometabolic disease. To be diagnosed with metabolic syndrome, you must have at least 2 of the 6 following risk factors:

- Overweight or obesity
- Increased waist circumference (abdominal obesity)
- Increased triglycerides or receiving treatment for elevated triglycerides
- Increased blood pressure or receiving treatment for elevated blood pressure
- Increased fasting glucose or receiving treatment for elevated blood glucose
- Decreased high density lipoprotein cholesterol (HDL-C) or receiving treatment for low HDL-C

**Do I have to take part?**

It is up to you to decide whether to take part in this study. If after reading this information sheet, you do decide to take part, you will be asked to sign a consent form. You may withdraw from the study at any time without giving a reason. Your choice will not affect the care that you receive from your general practitioner (GP), doctor or nurse.

**What will happen to me if I take part?**

At an initial screening for eligibility, you will be contacted by telephone or email to answer some questions to establish if you are likely to be eligible to take part. These questions will include general information about your health. If you are eligible and decide to take part, you will be invited to come to the University of Leeds for a baseline examination.

For the baseline examination, you will be asked to come to the University of Leeds **fasted**. Your last meal should before 10pm the night before coming to the University. You will be asked not to drink alcohol the night before or do any strenuous exercise the night before or the morning of screening examination. You will need to have signed the research study consent form before the exam. You will receive a copy of the signed consent form and this information sheet. You will have your height, weight, waist circumference and blood pressure measured, and be asked do a finger prick blood test to test your blood fasting glucose, high density lipoprotein cholesterol (HDL-C, ‘good cholesterol’) and triglycerides in your blood. These measurements will help us to confirm that you have metabolic syndrome. You will be instantly informed of your results and if eligible, you will be invited to take part in the intervention study. Once confirmed you are eligible, we will draw blood from your arm, for analysis of indicators of oxidative stress, inflammation, liver function and other metabolites. This will be done by a trained specialist—a phlebotomist— and only a small amount of blood will be taken (15 ml). At the end, you will then be given a total amount of supplements for 6 weeks. Then you will be informed with a date to come to the University after 6 weeks. At the following visit, you will again have your weight, waist circumference and blood pressure measured, and have a finger prick blood test for fasting glucose, triglycerides and HDL tests. We also will draw blood from your arm.

The supplementation period will last for 12 weeks. You will collect your supplements twice – once at the beginning of the period (or baseline), once at 6 weeks. During the 12-week supplementation period, you will be asked to take one capsule daily at approximately the same time daily (e.g. before you brush your teeth in the morning to help remember). We will ask you to come back to the University at 6 weeks and the end of study (12 weeks) bringing the supplement package and any untaken capsules with you. We will repeat the measurements: height, weight, waist circumference, blood pressure, fasting glucose, triglycerides and HDL-C, and the blood draw from your arm at 6 weeks and 12 weeks. You will be asked to avoid high mushroom intake during the supplementation period.

**Due to the current COVID-19 epidemic, you are required to wear a face covering during the visit. You are required to inform us and cancel the visit if you have any of the covid symptoms (fever, coughing, short of breath, loss of taste and/or smell), and/or if you have tested positive for COVID-19, and/or if you live with anyone who has tested positive for COVID-19. We will phone you at least 1 hour before you come to the University to confirm your health on the day. If you can come to the University, before we start sample collection, your body temperature will be taken with a non-contact temperature monitor at the entrance to the Chemistry building on Woodhouse Lane. Once normal body temperature is confirmed, you will be provided hand sanitiser to clean your hands and be escorted through the building following a designated one way system. You will also be escorted back out of the building. You are required to inform us if you develop any of the covid symptoms within the 48 hours (2 days) after the visit.**

**What are the possible disadvantages and risks of taking part?**

We believe the risks to taking part are very low. Ergothioneine is an amino acid normally found in food (it is found in high amounts in mushrooms) and the doses of ergothioneine being tested (5 or 30 mg/day) are well below the safety limit (800 mg/kg bodyweight per day—e.g. if you weigh 70kg, you could safely consume up to a total of 56,000mg per day). Ergothioneine has minimal allergy risk determined by European Food Safety Authority. While there might be a small discomfort when having your blood drawn, this will be only momentary.

**Due to the close contact requirements for obtaining blood samples and the current COVID-19 epidemic in circulation, there is risk of potential COVID transmission. All of our researchers will wear appropriate PPEs, including a face mask along with a face shield, single use gloves and a single use apron. All researchers will have their body temperature measured each day (being normal) and will be regularly tested for covid-19 (negative results)to reduce risk to of COVID transmission. The study desks and chairs will be cleaned down before and after the visit. There will be minimum of 1 hour between participants and the room will be aired. The window will also be opened during the visit. Should a participant report COVID-19 symptoms within 48 hours of visiting the University, the researchers who met them will immediately self-isolate and any other participants who interacted with the researchers within that timespan will be notified.**

**What are the possible benefits of taking part?**

Ergothioneine may be beneficial to people with metabolic syndrome. The information from this study will help us to learn if taking ergothioneine as a supplement can reduce risks and/or symptoms of metabolic syndrome, oxidative damage, inflammation and liver function. Your risk factors will be measured several times and the results shared with you. If our study does conclude a benefit to taking ergothioneine we will recommend all participants to take it at the end of the study.

You will receive a voucher from amazon or on your request at the end of the study. It will cover your travel fees to the University of Leeds (3 times) and an additional £10 for a small thank you.

**Will the information be kept confidential?**

Yes, all the collected information will be kept confidential. The personal information collected from participants will be coded and cannot be identified by any other people apart from research team members. All the data about participants’ personal information will be encrypted or locked at the University of Leeds.

**What will happen to the results of the study?**

All the contact information that we collect about you during the study will be kept strictly confidential and will stored separately from the research data. Only anonymised data will be used in the data analysis and publications.

The results will be used in PhD dissertation, and may also be used in academic journal publications and academic conference presentation.

**Who is organising/ funding the research?**

This study is organised and funded by the University of Leeds.

**Who has reviewed this study?**

This study has been approved by the Faculty Research Ethics Committee at The University of Leeds (**MEEC 20-007**).

**Thank you for taking the time to read this information.**

**If you have any questions or would like more information, please contact our research team:**

**Researcher:** Xiaoying Tian 07938500527 [ErgMS@leeds.ac.uk](mailto:ErgMS@leeds.ac.uk)

**Research supervisor:** Dr Bernadette Moore [J.B.Moore@leeds.ac.uk](mailto:J.B.Moore@leeds.ac.uk)

School of Food Science and Nutrition, Faculty of Environment, University of Leeds, LS2 9JT


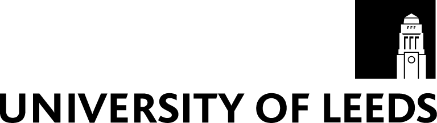


# Consent Form

**To take part in:** Investigating the potential health benefits of ergothioneine supplementation in people with metabolic syndrome (ErgMS)

| Please mark next to the statement if you agree: | | |
| --- | --- | --- |
| 1. I confirm that I have read and understood the information on the information sheet dated August 2021 v3.0 explaining the above research project. I have had the opportunity to consider the information and ask questions about the project. | Yes | No |
| 1. I understand that my participation is voluntary and that I am free to withdraw at any time without giving reasons, without my medical care or legal rights being affected. | Yes | No |
| 1. I understand that all the information about me, collected as part of this study, will be kept securely and that my personal details will not be available to anyone outside the research team. The use of a code instead of my name will guarantee the anonymity unless the code is broken for some safety reasons. | Yes | No |
| 1. I understand that the samples and/or information collected from/about me will be used to support other research in the future, and may be shared anonymously with other researchers. | Yes | No |
| 1. I agree to informing my General Practitioner of my participation in this study, if necessary. | Yes | No |
| 1. I agree to take part in the above research project and will inform the lead researcher when my contact details change. | Yes | No |
| 1. I agree to wear a face mask during the visit and to notify the researchers if I develop any COVID-19 symptoms and/or get positive result. | Yes | No |
| 1. Please send me a copy of the results of the above study. | Yes | No |
| 1. I agree I may be contacted to support other research in the future, and that my contact details may be used by other researchers from the University of Leeds in order to invite me to participate in future research studies. (**OPTIONAL**) | Yes | No |

| Name of participant | Date |
| --- | --- |
| ________________________ | __________________ |

*When completed, one copy for participant, one copy for researcher site file.*

*If you have any questions, please feel free to contact: Ms Xiaoying Tian 07938500527* [*ErgMS@leeds.ac.uk*](mailto:ErgMS@leeds.ac.uk)

*School of Food Science and Nutrition, University of Leeds, LS2 9J*


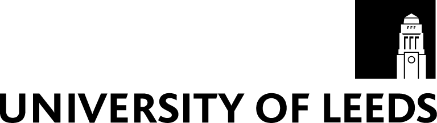


# Primary Eligibility Questionnaire

Thank you for your interest in taking part in this study to investigate the effects of ergothioneine on markers of metabolic syndrome, oxidative stress, inflammation and liver function.

To help determine whether you are eligible to take part, we need to take some general information from you and ask some questions about your lifestyle and health.

| **Contact details** | |
| --- | --- |
| First name |  |
| Surname |  |
| Telephone number |  |
| Mobile number |  |
| Email address |  |
| Home address |  |

| **General information** | | |
| --- | --- | --- |
| Gender | Male | Female |
| Date of birth |  | |
| Height | (cm / metres / feet & inches) | |
| Weight | (kg / lbs / stones & lbs) | |
| Waist circumference | (cm / feet & inches) | |
| Note: this is a measurement around the fullest point of your waist. If you do not have a tape, please write your dress/trousers size starting with ‘dress size:’ or ‘trousers size:’. | | |

| **Lifestyle** | | | |
| --- | --- | --- | --- |
| Are you pregnant, lactating or trying to conceive? | | YES | NO |
| Do you smoke? | | YES | NO |
| Do you drink alcohol? | | YES | NO |
| **If yes,** how much do you drink alcohol per week? | |  | |
| As a guide, 1 pint of beer / 1½ bottles of lager / 1 medium (175 mL) glass of wine = 2-3 units | | | |
| Have you taken or are you currently taking any vitamin or herbal supplements? | | YES | NO |
| **if yes**, please give details. Which supplements, how often, how many times a week or month?  If not currently, when was the last time you took a supplement? |  | | |
| Have you gained or lost weight more than 2-3kg in last 3 months? | | YES | NO |
| Are you currently on a specific diet or trying to lose weight? | | YES | NO |
| Do you regularly exercise? | | YES | NO |
| **If yes,** how vigorously, how often and how long? |  | | |
| Are you currently following advice to change your lifestyle (e.g. newly taking up exercise)? | | YES | NO |

| **Health condition** | | | | | | |
| --- | --- | --- | --- | --- | --- | --- |
| Have you had a covid vaccine? | | | YES | | NO | |
| **If yes**, which type? | | |  | | | |
| **If yes**, how many doses have you had? | | | 1 | 2 | | Other |
| what date was last dose administered? | | |  | | | |
| Have you ever been told that you have any of the following? | | | | | | |
| High blood pressure (hypertension) | | | YES | | NO | |
| High blood triglycerides | | | YES | | NO | |
| High blood glucose | | | YES | | NO | |
| Altered blood cholesterol (high LDL or low HDL) | | | YES | | NO | |
| Do you have any diseases in your family history? | | | YES | | NO | |
| **If yes**, please give details. | |  | | | | |
| Have you been diagnosed with any of the following? | | | | | | |
| Liver disease | | | YES | | NO | |
| **If yes**, please give details. |  | | | | | |
| Diabetes | | | YES | | NO | |
| Heart disease | | | YES | | NO | |
| Intestinal disorders (Crohn’s disease, short bowel syndrome, pancreatic insufficiency, cystic fibrosis, tropical sprue, whipple’s disease, chronic pancreatitis, gastrojejunostomy, surgical treatment for obesity, cholestasis, biliary atresia, parasite infections) | | | YES | | NO | |
| Kidney disease | | | YES | | NO | |
| Blood borne disease (HepB, HIV etc.) | | | YES | | NO | |
| Cancer | | | YES | | NO | |
| **If yes**, when did treatment end? | | |  | | | |
| Any other disease in the last 2 years? | | | YES | | NO | |
| **If yes**, please give details. | | |  | | | |
| Are you currently taking or regularly taking any prescription or non-prescription medicine? | | | YES | | NO | |
| **If yes**, please give details**.** | |  | | | | |
| Are you taking medication for diabetes or high blood pressure? | | | YES | | NO | |
| Have you ever had antibiotic treatment? | | | YES | | NO | |
| **If yes**, what is the first time you receiving the treatment?  When did treatment end? | | |  | | | |

| **Ability** | | |
| --- | --- | --- |
| Are you currently taking part in any other clinical trial? | YES | NO |
| Are you able to come to the University of Leeds on 3 time points during the 12 weeks (before start, 6 weeks, 12 weeks)? | YES | NO |
| Are you able to make your own decisions and **not** under the care of the state, by judicial or administrative decision? | YES | NO |

Thank you for answering these questions.


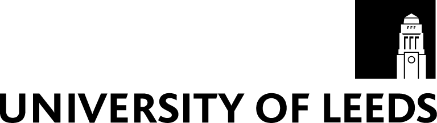


# Visit 1 Baseline Clinical Research File

| **Contact details** | |
| --- | --- |
| Screening ID |  |
| First name |  |
| Surname |  |
| Telephone number |  |
| Mobile number |  |
| Email address |  |
| Home address |  |

| **General information** | | |
| --- | --- | --- |
| Gender | Male | Female |
| Date of birth (age) |  | |
| Ethnicity | Asian/Asian British | White and all other ethnic groups |

| Date & Time of examination: _______________________________________________ | | | |
| --- | --- | --- | --- |
| **Physical measurement** | Measurements | Fits MetS Criteria? (Y/ N) | MetS Criteria |
| Height (cm) |  |  |  |
| Weight (kg) |  |  |  |
| Waist circumference (cm) |  |  | **Asian/Asian British:**  Men ≥ 90 cm; Women ≥ 80 cm;  **White and all other ethnic groups:** Men ≥ 94 cm, Women ≥ 80 cm |
| Systolic BP (mm Hg) |  |  | ≥ 130 mm Hg |
| Diastolic BP (mm Hg) |  |  | ≥ 85 mm Hg |

| Date & Time of blood collection: _______________________________________________ | | | |
| --- | --- | --- | --- |
| **Blood analysis** | Concentration | Fits MetS Criteria? (Y/N) | MetS Criteria |
| Triglycerides (mg/dL) |  |  | ≥ 150 mg/dL |
| HDL-cholesterol (mg/dL) |  |  | Male < 40 mg/dL Female < 50 mg/dL |
| Glucose (mmol/L) |  |  | ≥ 5.6 mmol/L |

| MetS criteria check (please tick √ which apply) | | | | | |
| --- | --- | --- | --- | --- | --- |
| Waist Circumference | Blood Pressure (SBP and/or DBP) | Triglycerides | HDL-cholesterol | Glucose | No. of MetS criteria met |
|  |  |  |  |  |  |

**End of Visit 1 Baseline CRF**


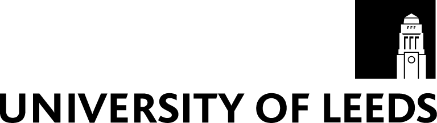


# Visit 2 Follow-up 6 Weeks Clinical Research File

| Screening ID |  |
| --- | --- |

| Number of untaken tablets: |  |
| --- | --- |

| Date & Time of examination: _______________________________________________ | |  |
| --- | --- | --- |
| **Physical measurement** | Measurements | |
| Height (cm) |  | |
| Weight (kg) |  | |
| Waist circumference (cm) |  | |
| Systolic BP (mm Hg) |  | |
| Diastolic BP (mm Hg) |  | |

| Date & Time of blood collection: _______________________________________________ | |  |
| --- | --- | --- |
| **Blood analysis** | Concentration | |
| Triglycerides (mg/dL) |  | |
| HDL-cholesterol (mg/dL) |  | |
| Glucose (mmol/L) |  | |

**End of Visit 2 Follow-up 6 Weeks CRF**


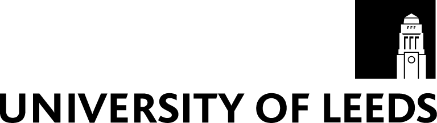


# Visit 3 Endpoint Clinical Research File

| Screening ID |  |
| --- | --- |

| Number of untaken tablets: |  |
| --- | --- |

| Date & Time of examination: _______________________________________________ | |  |
| --- | --- | --- |
| **Physical measurement** | Measurements | |
| Height (cm) |  | |
| Weight (kg) |  | |
| Waist circumference (cm) |  | |
| Systolic BP (mm Hg) |  | |
| Diastolic BP (mm Hg) |  | |

| Date & Time of blood collection: _______________________________________________ | |  |
| --- | --- | --- |
| **Blood analysis** | Concentration | |
| Triglycerides (mg/dL) |  | |
| HDL-cholesterol (mg/dL) |  | |
| Glucose (mmol/L) |  | |

**End of Visit 3 Endpoint CRF**


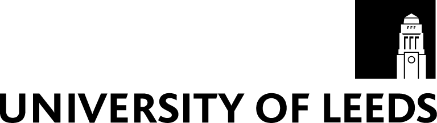


# Food Frequency Questionnaire

The following questions ask about some foods and drinks you might have during a 'typical week', over the past month or so. Do not be concerned if some things you eat or drink are not mentioned.

Please tick how often you eat at least ONE portion of the following foods and drinks: (a portion includes: a handful of grapes, an orange, a side salad, a slice of bread, a medium potato, half a pint glass of soft drink)

(Please only put one tick, but answer **EVERY** line)

|  | | **Rarely or never** | **Less than 1 a week** | **Once a week** | **2-3 times a week** | **4-6 times a week** | **1-2 times a day** | **3-4 times a day** | **5+ a day** |
| --- | --- | --- | --- | --- | --- | --- | --- | --- | --- |
| 1. | Fruit - fresh and/or frozen | ◻ | ◻ | ◻ | ◻ | ◻ | ◻ | ◻ | ◻ |
| 2. | Fruit juice/ smoothie (not cordial or squash) | ◻ | ◻ | ◻ | ◻ | ◻ | ◻ | ◻ | ◻ |
| 3. | Jam or marmalade | ◻ | ◻ | ◻ | ◻ | ◻ | ◻ | ◻ | ◻ |
| 4. | Salad or raw vegetables | ◻ | ◻ | ◻ | ◻ | ◻ | ◻ | ◻ | ◻ |
| 5. | Vegetables - cooked (tinned/frozen/fresh but not potatoes) | ◻ | ◻ | ◻ | ◻ | ◻ | ◻ | ◻ | ◻ |
|  | | | | | | | | | |
| 6. | Potatoes inc skin (steamed, boiled or baked) | ◻ | ◻ | ◻ | ◻ | ◻ | ◻ | ◻ | ◻ |
| 7. | Potatoes no skin (steamed, boiled or mashed) | ◻ | ◻ | ◻ | ◻ | ◻ | ◻ | ◻ | ◻ |
| 8. | Potatoes (fried or roasted in fat or oil) | ◻ | ◻ | ◻ | ◻ | ◻ | ◻ | ◻ | ◻ |
| 9. | Beans/pulses/legumes (inc. baked beans, chickpeas, dahl, lentils, etc.) | ◻ | ◻ | ◻ | ◻ | ◻ | ◻ | ◻ | ◻ |
| 10. | White bread or bread rolls | ◻ | ◻ | ◻ | ◻ | ◻ | ◻ | ◻ | ◻ |
| 11. | Brown, sourdough, spelt, or rye bread or bread rolls | ◻ | ◻ | ◻ | ◻ | ◻ | ◻ | ◻ | ◻ |
| 12. | Breakfast cereal - High fibre (inc. weetabix, fruit & fibre, porridge or musili) | ◻ | ◻ | ◻ | ◻ | ◻ | ◻ | ◻ | ◻ |
| 13. | Breakfast cereal - NOT high fibre (cornflakes, rice crispies, coco pops, ect.) | ◻ | ◻ | ◻ | ◻ | ◻ | ◻ | ◻ | ◻ |
| 14. | White rice or pasta | ◻ | ◻ | ◻ | ◻ | ◻ | ◻ | ◻ | ◻ |
| 15. | Brown, whole grain rice or pasta | ◻ | ◻ | ◻ | ◻ | ◻ | ◻ | ◻ | ◻ |
|  | | | | | | | | | |
| 16. | Soft drinks - made with sugar (fizzy drinks i.e. Coca Cola/lemonade) | ◻ | ◻ | ◻ | ◻ | ◻ | ◻ | ◻ | ◻ |
| 17. | Soft drinks - sugar free (diet drinks and squashes) | ◻ | ◻ | ◻ | ◻ | ◻ | ◻ | ◻ | ◻ |
| 18. | Sweets, cakes, scones, pancakes, biscuits and chocolate | ◻ | ◻ | ◻ | ◻ | ◻ | ◻ | ◻ | ◻ |
| 19. | Pudding or dessert inc. ice cream | ◻ | ◻ | ◻ | ◻ | ◻ | ◻ | ◻ | ◻ |
| 20. | Crisps, savoury snacks (crackers, pringles, tortilla chips, crisps & dips) | ◻ | ◻ | ◻ | ◻ | ◻ | ◻ | ◻ | ◻ |
|  | | | | | | | | | |
| 21. | Tea - black/green or herbal | ◻ | ◻ | ◻ | ◻ | ◻ | ◻ | ◻ | ◻ |
| 22. | Instant coffee/americano/filter | ◻ | ◻ | ◻ | ◻ | ◻ | ◻ | ◻ | ◻ |
| 23. | Coffee drink (latte, cappuccino, hot chocolate, with syrup) | ◻ | ◻ | ◻ | ◻ | ◻ | ◻ | ◻ | ◻ |
| 24. | Coffee drink (latte, cappuccino without syrup) | ◻ | ◻ | ◻ | ◻ | ◻ | ◻ | ◻ | ◻ |
| 25. | Sugar added to tea/coffee or other foods (i.e. cereal or in cooking) | ◻ | ◻ | ◻ | ◻ | ◻ | ◻ | ◻ | ◻ |
| 26. | Sweeteners added to food/drinks | ◻ | ◻ | ◻ | ◻ | ◻ | ◻ | ◻ | ◻ |
|  | | | | | | | | | |
| 27. | Red meat: beef, lamb, pork, ham, steaks, joints for roasting, mince or chops | ◻ | ◻ | ◻ | ◻ | ◻ | ◻ | ◻ | ◻ |
| 28. | White meat: chicken or turkey; steaks, roast, whole bird, mince or portions (not in batter or bread crumbs) | ◻ | ◻ | ◻ | ◻ | ◻ | ◻ | ◻ | ◻ |
| 29. | Processed red meat: sausages, burgers, bacon, corned beef, pasties/pies | ◻ | ◻ | ◻ | ◻ | ◻ | ◻ | ◻ | ◻ |
| 30. | Processed white meat: nuggets, twizlers, burgers, pies, anything in batter or breadcrumbs | ◻ | ◻ | ◻ | ◻ | ◻ | ◻ | ◻ | ◻ |
| 31. | Fish in batter or bread crumbs (i.e. fried fish) | ◻ | ◻ | ◻ | ◻ | ◻ | ◻ | ◻ | ◻ |
| 32. | White fish (not in batter or breadcrumbs) | ◻ | ◻ | ◻ | ◻ | ◻ | ◻ | ◻ | ◻ |
| 33. | Oily fish: herring, sardines, salmon, trout, mackerel, fresh tuna (not tinned tuna or salmon) | ◻ | ◻ | ◻ | ◻ | ◻ | ◻ | ◻ | ◻ |
| 34. | Eggs - poached, boiled, scrambled, etc. | ◻ | ◻ | ◻ | ◻ | ◻ | ◻ | ◻ | ◻ |
| 35. | Nuts and Seeds (all types) | ◻ | ◻ | ◻ | ◻ | ◻ | ◻ | ◻ | ◻ |
|  | | | | | | | | | |
| 36. | Dairy - flavoured yoghurt (low or full fat) | ◻ | ◻ | ◻ | ◻ | ◻ | ◻ | ◻ | ◻ |
| 37. | Dairy - natural yoghurt; no flavour (low or full fat) | ◻ | ◻ | ◻ | ◻ | ◻ | ◻ | ◻ | ◻ |
| 38. | Dairy - all cheese | ◻ | ◻ | ◻ | ◻ | ◻ | ◻ | ◻ | ◻ |
| 39. | Fats used as spread or for cooking (lard, butter, coconut oil or solid margarine) | ◻ | ◻ | ◻ | ◻ | ◻ | ◻ | ◻ | ◻ |
| 40. | Fats used as spread or for cooking (olive oil, vegetable oil, low fat sprays, heart healthy spreads (Benecol)) | ◻ | ◻ | ◻ | ◻ | ◻ | ◻ | ◻ | ◻ |
| 41. | Spreads - nut butter (peanut, almond, ect.), chocolate spread | ◻ | ◻ | ◻ | ◻ | ◻ | ◻ | ◻ | ◻ |
| 42. | Salad dressing: salad cream, mayonnaise, oil based dressing | ◻ | ◻ | ◻ | ◻ | ◻ | ◻ | ◻ | ◻ |
| 43. | Nuts and Seeds (all types) | ◻ | ◻ | ◻ | ◻ | ◻ | ◻ | ◻ | ◻ |
|  | | | | | | | | | |
| 44. | Fried foods & take away foods (Indian, pizza, chinese, fish and chips, noodles, etc.) | ◻ | ◻ | ◻ | ◻ | ◻ | ◻ | ◻ | ◻ |
|  |  |  |  |  |  |  |  |  |  |
|  |  | **Full Fat Cows** | **Semi-Skimmed Cows Milk** | | **Skimmed Cows Milk** | | **No Milk** | **Other Milk (Inc soy, almond, ect.)** | |
| 44. | Specify type of milk most frequently consumed | ◻ | ◻ | | ◻ | | ◻ | ◻ | |
|  |  |  |  |  |  |  |  |  |  |
|  |  | **Never** | | **Rarely (< 4 Standard Drinks*)** | | **Occasionally (< 12 Standard Drinks*)** | | **Frequently (>12 Standard Drinks*)** | |
| 45 | Approximately how often do you drink alcohol per week? | ◻ | | ◻ | | ◻ | | ◻ | |
| *A standard drink is shown in the image below.  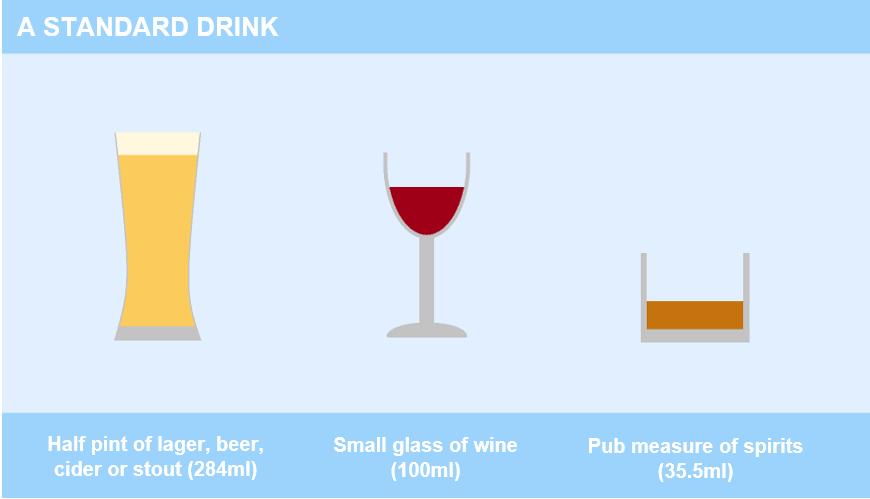 | | | | | | | | | |


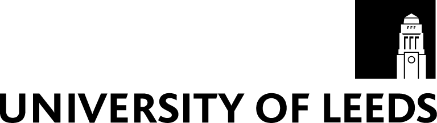


# Food Frequency Questionnaire (Erg focused)

| *The following questions ask about some foods high in ergothioneine and how many portions you might have during a 'typical week', over the past six weeks or so. Do not be concerned if some things you eat or drink are not mentioned.   Please tick how often you eat at least ONE portion of the following foods. Note 1 portion = 80g including dried, fresh, cooked, canned and processed food. For mushrooms this is typically about 14 button mushrooms, or 3-4 heaped tablespoons of cooked mushrooms or 2 tablespoons if dried.  Please only put one tick, but answer EVERY line.* | | | | | | | | |  |
| --- | --- | --- | --- | --- | --- | --- | --- | --- | --- |
|  |  |  |  |  |  |  |  |  |  |
|  |  |  |  |  |  |  |  |  |  |
|  |  |  |  |  |  |  |  |  |  |
|  |  |  |  |  |  |  |  |  |  |
|  |  |  |  |  |  |  |  |  |  |
|  |  |  |  |  |  |  |  |  |  |
|  |  |  |  |  |  |  |  |  |  |
|  |  |  |  |  |  |  |  |  |  |
|  |  |  |  |  |  |  |  |  |  |
| **MUSHROOMS** | **Rarely or never** | **Less than 1 a week** | **Once a week** | **2-3 times a week** | **4-6 times a week** | **1-2 times a day** | **3-4 times a day** | **5+ a day** |  |
| Abalone | ◻ | ◻ | ◻ | ◻ | ◻ | ◻ | ◻ | ◻ |  |
| Boletus edulis | ◻ | ◻ | ◻ | ◻ | ◻ | ◻ | ◻ | ◻ |  |
| Button-brown | ◻ | ◻ | ◻ | ◻ | ◻ | ◻ | ◻ | ◻ |  |
| Button-white | ◻ | ◻ | ◻ | ◻ | ◻ | ◻ | ◻ | ◻ |  |
| Enoki | ◻ | ◻ | ◻ | ◻ | ◻ | ◻ | ◻ | ◻ |  |
| Fungus-black | ◻ | ◻ | ◻ | ◻ | ◻ | ◻ | ◻ | ◻ |  |
| Fungus-white | ◻ | ◻ | ◻ | ◻ | ◻ | ◻ | ◻ | ◻ |  |
| Golden oyster | ◻ | ◻ | ◻ | ◻ | ◻ | ◻ | ◻ | ◻ |  |
| King oyster | ◻ | ◻ | ◻ | ◻ | ◻ | ◻ | ◻ | ◻ |  |
| Maitake | ◻ | ◻ | ◻ | ◻ | ◻ | ◻ | ◻ | ◻ |  |
| Oyster | ◻ | ◻ | ◻ | ◻ | ◻ | ◻ | ◻ | ◻ |  |
| Portobello | ◻ | ◻ | ◻ | ◻ | ◻ | ◻ | ◻ | ◻ |  |
| Shiitake | ◻ | ◻ | ◻ | ◻ | ◻ | ◻ | ◻ | ◻ |  |
| Shimeji | ◻ | ◻ | ◻ | ◻ | ◻ | ◻ | ◻ | ◻ |  |
| Willow | ◻ | ◻ | ◻ | ◻ | ◻ | ◻ | ◻ | ◻ |  |
| Wood ear | ◻ | ◻ | ◻ | ◻ | ◻ | ◻ | ◻ | ◻ |  |
| Other(s) | ◻ | ◻ | ◻ | ◻ | ◻ | ◻ | ◻ | ◻ |  |
| **ORGAN MEATS** | **Rarely or never** | **Less than 1 a week** | **Once a week** | **2-3 times a week** | **4-6 times a week** | **1-2 times a day** | **3-4 times a day** | **5+ a day** |  |
| Chicken liver | ◻ | ◻ | ◻ | ◻ | ◻ | ◻ | ◻ | ◻ |  |
| Pork liver | ◻ | ◻ | ◻ | ◻ | ◻ | ◻ | ◻ | ◻ |  |
| Any liver pate | ◻ | ◻ | ◻ | ◻ | ◻ | ◻ | ◻ | ◻ |  |
| Pork kidney | ◻ | ◻ | ◻ | ◻ | ◻ | ◻ | ◻ | ◻ |  |
| Haggis | ◻ | ◻ | ◻ | ◻ | ◻ | ◻ | ◻ | ◻ |  |
| **Tempeh** | ◻ | ◻ | ◻ | ◻ | ◻ | ◻ | ◻ | ◻ |  |

**End of Questionnaire**

**Thank you for your time answering these questions!**


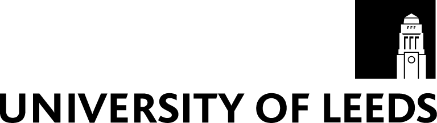


# Visit 1 Health Questionnaire

| Screening ID |  |
| --- | --- |

| Have you had a covid vaccine? | | YES | | | NO | |
| --- | --- | --- | --- | --- | --- | --- |
| **If yes**, which type? | |  | | | | |
| **If yes**, how many doses have you had? | | 1 | 2 | | | Other |
| what date was last dose administered? | |  | | | | |
| Are you pregnant, lactating or trying to conceive? | | YES | | NO | | |
| Do you smoke? | | YES | | NO | | |
| Do you drink alcohol? | | YES | | NO | | |
| **If yes,** how much do you drink alcohol per week? | |  | | | | |
| As a guide, 1 pint of beer / 1½ bottles of lager / 1 medium (175 mL) glass of wine = 2-3 units | | | | | | |
| Have you taken or are you currently taking any vitamin or herbal supplements? | | YES | | NO | | |
| **if yes**, how often/how many times a week or month?  If not currently, when was the last time you took a supplement? |  | | | | | |
| **If yes**, please give details. What supplements/ how often. |  | | | | | |
| Have you gained or lost weight more than 2-3kg in last 3 months? | | YES | | NO | | |
| Are you currently on a specific diet or trying to lose weight? | | YES | | NO | | |
| Do you regularly exercise? | | YES | | NO | | |
| **If yes,** how vigorously, how often and how long? |  | | | | | |
| Are you currently following advice to change your lifestyle (e.g. newly taking up exercise)? | | YES | | NO | | |

| Have you ever been told that you have any of the following? | | | | |
| --- | --- | --- | --- | --- |
| High blood pressure (hypertension) | | | YES | NO |
| High blood triglycerides | | | YES | NO |
| High blood glucose | | | YES | NO |
| Altered blood cholesterol (high LDL or low HDL) | | | YES | NO |
| Have you been diagnosed with any of the following? | | | | |
| Liver disease | | | YES | NO |
| **If yes**, please give details. |  | | | |
| Diabetes | | | YES | NO |
| Heart disease | | | YES | NO |
| Intestinal disorders (Crohn’s disease, short bowel syndrome, pancreatic insufficiency, cystic fibrosis, tropical sprue, whipple’s disease, chronic pancreatitis, gastrojejunostomy, surgical treatment for obesity, cholestasis, biliary atresia, parasite infections) | | | YES | NO |
| Kidney disease | | | YES | NO |
| Cancer | | | YES | NO |
| **If yes**, when did treatment end? | | |  | |
| Any other disease in the last 2 years? | | | YES | NO |
| **If yes**, please give details. | | |  | |
| Are you currently taking or regularly taking any prescription or non-prescription medicine? | | | YES | NO |
| **If yes**, please give details**.** | |  | | |
| Are you taking medication for diabetes or high blood pressure? | | | YES | NO |
| Have you had antibiotic treatment? | | | YES | NO |
| **If yes**, what is the first time you receiving the treatment?  When did treatment end? | | |  | |


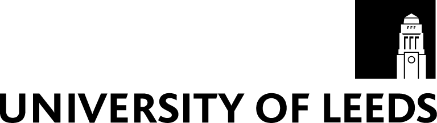


# Visit 2 Health Questionnaire

| Screening ID |  |
| --- | --- |

| Have you had a covid vaccine? | | YES | | | NO | |
| --- | --- | --- | --- | --- | --- | --- |
| **If yes**, which type? | |  | | | | |
| **If yes**, how many doses have you had? | | 1 | 2 | | | Other |
| what date was last dose administered? | |  | | | | |
| Are you pregnant, lactating or trying to conceive? | | YES | | NO | | |
| Do you smoke? | | YES | | NO | | |
| Do you drink alcohol? | | YES | | NO | | |
| **If yes,** how much do you drink alcohol per week? | |  | | | | |
| As a guide, 1 pint of beer / 1½ bottles of lager / 1 medium (175 mL) glass of wine = 2-3 units | | | | | | |
| Have you taken or are you currently taking any vitamin or herbal supplements? | | YES | | NO | | |
| **if yes**, how often/how many times a week or month?  If not currently, when was the last time you took a supplement? |  | | | | | |
| **If yes**, please give details. What supplements/ how often. |  | | | | | |
| Have you gained or lost weight more than 2-3kg in last 3 months? | | YES | | NO | | |
| Are you currently on a specific diet or trying to lose weight? | | YES | | NO | | |
| Do you regularly exercise? | | YES | | NO | | |
| **If yes,** how vigorously, how often and how long? |  | | | | | |
| Are you currently following advice to change your lifestyle (e.g. newly taking up exercise)? | | YES | | NO | | |

| Have you ever been told that you have any of the following? | | | | |
| --- | --- | --- | --- | --- |
| High blood pressure (hypertension) | | | YES | NO |
| High blood triglycerides | | | YES | NO |
| High blood glucose | | | YES | NO |
| Altered blood cholesterol (high LDL or low HDL) | | | YES | NO |
| Have you been diagnosed with any of the following? | | | | |
| Liver disease | | | YES | NO |
| **If yes**, please give details. | |  | | |
| Diabetes | | | YES | NO |
| Heart disease | | | YES | NO |
| Intestinal disorders (Crohn’s disease, short bowel syndrome, pancreatic insufficiency, cystic fibrosis, tropical sprue, whipple’s disease, chronic pancreatitis, gastrojejunostomy, surgical treatment for obesity, cholestasis, biliary atresia, parasite infections) | | | YES | NO |
| Kidney disease | | | YES | NO |
| Cancer | | | YES | NO |
| **If yes**, when did treatment end? | | |  | |
| Any other disease in the last 2 years? | | | YES | NO |
| **If yes**, please give details. | | |  | |
| Are you currently taking or regularly taking any prescription or non-prescription medicine? | | | YES | NO |
| **If yes**, please give details**.** |  | | | |
| Are you taking medication for diabetes or high blood pressure? | | | YES | NO |
| Have you had antibiotic treatment? | | | YES | NO |
| **If yes**, what is the first time you receiving the treatment?  When did treatment end? | | |  | |

| Have you suffered a condition during administration period? | | YES | NO |
| --- | --- | --- | --- |
| **If yes**, please give details. |  | | |
| Have you suffered a symptom after taking supplements? | | YES | NO |
| **If yes**, please give details. |  | | |


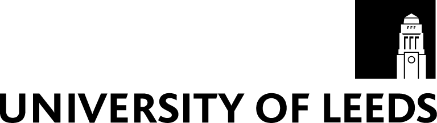


# Visit 3 Health Questionnaire

| Screening ID |  |
| --- | --- |

| Have you had a covid vaccine? | | YES | | | NO | |
| --- | --- | --- | --- | --- | --- | --- |
| **If yes**, which type? | |  | | | | |
| **If yes**, how many doses have you had? | | 1 | 2 | | | Other |
| what date was last dose administered? | |  | | | | |
| Are you pregnant, lactating or trying to conceive? | | YES | | NO | | |
| Do you smoke? | | YES | | NO | | |
| Do you drink alcohol? | | YES | | NO | | |
| **If yes,** how much do you drink alcohol per week? | |  | | | | |
| As a guide, 1 pint of beer / 1½ bottles of lager / 1 medium (175 mL) glass of wine = 2-3 units | | | | | | |
| Have you taken or are you currently taking any vitamin or herbal supplements? | | YES | | NO | | |
| **if yes**, how often/how many times a week or month?  If not currently, when was the last time you took a supplement? |  | | | | | |
| **If yes**, please give details. What supplements/ how often. |  | | | | | |
| Have you gained or lost weight more than 2-3kg in last 3 months? | | YES | | NO | | |
| Are you currently on a specific diet or trying to lose weight? | | YES | | NO | | |
| Do you regularly exercise? | | YES | | NO | | |
| **If yes,** how vigorously, how often and how long? |  | | | | | |
| Are you currently following advice to change your lifestyle (e.g. newly taking up exercise)? | | YES | | NO | | |

| Have you ever been told that you have any of the following? | | | | |
| --- | --- | --- | --- | --- |
| High blood pressure (hypertension) | | | YES | NO |
| High blood triglycerides | | | YES | NO |
| High blood glucose | | | YES | NO |
| Altered blood cholesterol (high LDL or low HDL) | | | YES | NO |
| Have you been diagnosed with any of the following? | | | | |
| Liver disease | | | YES | NO |
| **If yes**, please give details. |  | | | |
| Diabetes | | | YES | NO |
| Heart disease | | | YES | NO |
| Intestinal disorders (Crohn’s disease, short bowel syndrome, pancreatic insufficiency, cystic fibrosis, tropical sprue, whipple’s disease, chronic pancreatitis, gastrojejunostomy, surgical treatment for obesity, cholestasis, biliary atresia, parasite infections) | | | YES | NO |
| Kidney disease | | | YES | NO |
| Cancer | | | YES | NO |
| **If yes**, when did treatment end? | | |  | |
| Any other disease in the last 2 years? | | | YES | NO |
| **If yes**, please give details. | | |  | |
| Are you currently taking or regularly taking any prescription or non-prescription medicine? | | | YES | NO |
| **If yes**, please give details**.** | |  | | |
| Are you taking medication for diabetes or high blood pressure? | | | YES | NO |
| Have you had antibiotic treatment? | | | YES | NO |
| **If yes**, what is the first time you receiving the treatment?  When did treatment end? | | |  | |

| Have you suffered a condition during administration period? | | YES | NO |
| --- | --- | --- | --- |
| **If yes**, please give details. |  | | |
| Have you suffered a symptom after taking supplements? | | YES | NO |
| **If yes**, please give details. |  | | |

| Do you think which dose of ergothioneine you are taking during this period? | | |
| --- | --- | --- |
| Placebo | 5 mg/day | 30 mg/day |
| **Why?** |  | |


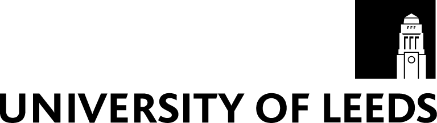


# Adverse Events /Serious Adverse Events Form

| Participant ID: |  |  |
| --- | --- | --- |

| Date of report: |  |  |
| --- | --- | --- |

| Name of reporter: |  |  |
| --- | --- | --- |

| Source of information: |  |
| --- | --- |

| Description of event: | | | |
| --- | --- | --- | --- |
|  | | | |
| Dates of event: | Start: | | |
|  | End: | | |
| Still ongoing: | | Yes | No |
| GP contacted? | | Yes | No |
| Any medication taken for this AE? | | Yes | No |
| **If yes,** please specify: |  | | |
| Causality likely to be trial related? | | Yes | No |
| **Reporter Action taken:** |  | | |
